# Are restudy decisions reactively altered by judgments of learning? Insights from related and unrelated word pairs

**DOI:** 10.3758/s13423-026-03030-2

**Published:** 2026-10-22

**Authors:** Franziska Ingendahl, Monika Undorf

**Affiliations:** Department of Psychology, Technical University of Darmstadt, Alexanderstr. 10, 64283 Darmstadt, Germany

**Keywords:** Metamemory, Memory, Judgments of learning, Reactivity, Restudy decisions

## Abstract

Recent research shows that predicting one’s own future memory during learning (immediate judgments of learning, JOLs) can enhance memory for related word pairs and reduce memory for unrelated word pairs. It is an open question, however, whether reactive effects of immediate JOLs on memory performance are related to changes in people’s study behavior. The present study addresses this gap by examining whether the requirement to provide JOLs alters restudy decisions. In two preregistered experiments (Experiment 1a: *N* = 140, Experiment 1b: *N* = 202), participants provided JOLs or did not provide JOLs during the study of related and unrelated word pairs, made restudy decisions for all studied word pairs, and completed a cued-recall test. Results revealed no reactivity for related word pairs but negative reactivity for unrelated word pairs. Importantly, restudy decisions for related and unrelated word pairs did not differ reliably depending on the requirement to provide JOLs. Overall, the present findings indicate that immediate JOLs may not have reactive effects on metacognitive control decisions and imply that changes in restudy decisions assessed during learning may not underlie negative reactivity of immediate JOLs for unrelated word pairs, thus questioning the adequacy of the changed-goal account as an explanation of detrimental effects of JOLs on memory performance.

**Supplementary Information:**

The online version contains supplementary material available at https://doi.org/10.3758/s13423-026-03030-2.

## Introduction

Metacognitive judgments are common tools for assessing what people know about their cognitions (Dunlosky et al., 2016). Whereas these judgments were assumed to be non-reactive measures of metacognition (Jordano & Touron, 2018), a growing body of research reveals that assessing metacognitive judgments can alter cognitive processes (Double & Birney, 2019). In particular, much research has focused on reactivity of *immediate judgments of learning* (JOLs; Arbuckle & Cuddy, 1969; Koriat, 1997) – predictions of one’s own memory during learning – to memory performance (for a meta-analysis, see Ingendahl et al., 2025). However, underlying mechanisms of JOL reactivity remain poorly understood (Ingendahl et al., 2025; Myers et al., 2025). The present study aimed to improve the understanding of these mechanisms by examining whether JOLs alter decisions about subsequent learning.

### Judgments of learning (JOL) reactivity

JOLs are commonly used as a measure of metacognitive monitoring (for a review, see Rhodes, 2016). However, recent research shows that predicting one’s memory can alter memory compared to studying without JOLs (e.g., Mitchum et al., 2016; Soderstrom et al., 2015). A recent meta-analysis (Ingendahl et al., 2025) reveals that soliciting JOLs alters memory to a small extent overall and highlights that the direction of reactivity differs depending on the conditions under which it is examined. Research has identified three distinct reactivity types – positive, negative, and no reactivity. *Positive reactivity* posits that JOLs enhance memory and has been observed for forward-related (e.g., Janes et al., 2018; Soderstrom et al., 2015), backward-related (e.g., Maxwell & Huff, 2022, 2023), identical (e.g., Chang & Brainerd, 2022; Halamish & Undorf, 2023), mediated (e.g., Chang & Brainerd, 2025; Maxwell & Huff, 2024), and category-cued word pairs (Rivers et al., 2023) in cued-recall tests, and several item types in recognition tests (e.g., Myers et al., 2020, 2025; Shi et al., 2023). In contrast, *negative reactivity* refers to soliciting JOLs impairing memory, observed for unrelated word pairs in cued-recall tests (e.g., Halamish & Undorf, 2023; Mitchum et al., 2016; Undorf et al., 2024) and inter-item relational processing (e.g., Zhao, Li, et al., 2023a, 2023b; Zhao, Yin, et al., 2023a, 2023b). Importantly, JOLs do not inevitably alter memory, demonstrated by *no reactivity* in free-recall (e.g., Zhao, Li, et al., 2023a, 2023b) and source-memory tests (Myers et al., 2025).

Several accounts aim to explain reactivity (e.g., Janes et al., 2018; Mitchum et al., 2016; Senkova & Otani, 2021; Soderstrom et al., 2015). Among the most prominent accounts are the *cue-strengthening account* (Soderstrom et al., 2015) explaining positive reactivity as a result of increased processing of cues, the *dual-task account* (Mitchum et al., 2016) proposing that negative reactivity stems from JOLs interfering with learning, and the *changed-goal account* (Mitchum et al., 2016) stating that reactivity is due to motivational changes in goals pursued during learning. Because the latter account is particularly relevant to this study, we focus on it and return to other accounts in the *General discussion*.

The changed-goal account takes a motivational perspective on explaining why JOLs enhance memory for related pairs and/or reduce memory for unrelated pairs. It proposes that learners making JOLs realize they will not recall all items, making them shift their goal from mastering all items to focusing on those they perceive as easier (e.g., related pairs) and neglecting those they perceive as more difficult (e.g., unrelated pairs). This goal change can result in positive reactivity for related pairs, negative reactivity for unrelated pairs, or both, depending on whether learners prioritize studying easier pairs, deprioritize studying more difficult pairs, or prioritize studying easier pairs at the expense of more difficult pairs. Several studies align with the changed-goal account. For example, Janes and colleagues (2018) observed no reactivity when studying was self-paced and recalling all pairs was more attainable, and found JOL reactivity only when pairs differed in perceived difficulty. Further, the changed-goal account explains why reactivity for weakly related pairs resembles that for strongly related pairs when studied alongside more difficult pairs (e.g., Ingendahl & Undorf, 2026a) but resembles that for unrelated pairs when studied alongside easier pairs (Soderstrom et al., 2015). Also, it is one of few accounts explaining the simultaneous occurrence of positive and negative reactivity (e.g., Halamish & Undorf, 2023; Ingendahl & Undorf, 2026a; Li et al., 2024; Witherby et al., 2023). Other studies, however, question the changed-goal account by showing that reactivity can occur even in the absence of difficulty differences (Maxwell & Huff, 2023) and that prestudy JOLs are not reactive (e.g., Li et al., 2024).

Whereas previous tests of the changed-goal account focused on reactivity in memory, the present study tests its main proposition – that JOLs alter the goal pursued during learning – by examining the effect of JOLs on metacognitive control decisions.

### JOL reactivity and metacognitive control

According to Nelson and Narens’ (1990) metamemory model, people’s cognition and metacognition are connected by two processes: monitoring and control. Monitoring refers to learners’ assessments of their learning and is commonly examined with metacognitive judgments such as JOLs, whereas control describes learners’ regulation of their learning behavior and is usually assessed by behavior-oriented measures such as self-paced study time allocation (e.g., Thiede & Dunlosky, 1999) or restudy decisions (e.g., Nelson et al., 1994). Given that monitoring and control are closely intertwined (e.g., Koriat et al., 2006; Metcalfe & Finn, 2008), reactivity of JOLs may also alter control processes.

Although changes in learning behavior due to making JOLs are at the heart of the changed-goal account, little is known about whether assessing JOLs alters metacognitive control processes. Hinting toward a role of control processes in reactivity, Janes and colleagues (2018) found that JOLs altered memory performance for related and unrelated word pairs under experimenter-paced but not self-paced study conditions, implying that whether learners can control their learning may be crucial for JOL reactivity in memory. Further, Mitchum and colleagues (2016) observed that self-paced study times were less strongly related to difficulty when learners provided JOLs, suggesting that making JOLs impacted study time allocation. However, later studies examining JOL reactivity under self-paced study conditions (e.g., Double, 2023; Maxwell & Huff, 2022, 2023) rarely focused on the impact of JOLs on study times. Additionally, recent findings on strategy changes during learning showing that JOLs alter the strategies learners use for word-pair (Ingendahl & Undorf, 2026a, 2026b) and category learning (Double et al., 2026) suggest that soliciting JOLs could alter metacognitive control processes.

An underexplored approach to testing whether JOLs alter metacognitive control is to examine *restudy decisions* during learning (e.g., Nelson et al., 1994). Restudy decisions ask learners to select items for restudy to improve their performance on a later memory test (Finley et al., 2010). Established models of metacognitive control (e.g., the discrepancy reduction model, Dunlosky & Hertzog, 1998; and the region of proximal learning model, Metcalfe & Kornell, 2003) propose that learners base restudy decisions on their monitoring and preferably select items they have not mastered yet, which are typically of moderate or high difficulty. Supporting this prediction, it has been found that learners, for example, select abstract words more frequently than concrete words (e.g., Tauber & Rhodes, 2012), lesser-known topics more frequently than better-known topics (Shanks & Serra, 2014), and difficult pairs more frequently than easy pairs (e.g., Thiede & Dunlosky, 1999). Because restudy decisions directly assess metacognitive control, they provide a proximal indicator of learners’ goals and are well suited for testing the changed-goal account.

### Present study

The present study examined the consequences of soliciting JOLs to metacognitive control by examining restudy decisions. Specifically, this study tested whether soliciting JOLs alters restudy decisions as predicted by the changed-goal account. In two preregistered experiments, participants studied related and unrelated word pairs, provided JOLs during learning or studied the pairs without JOLs, and subsequently made a restudy decision for each pair before completing a cued-recall test. Experiments differed in the number of studied pairs, with Experiment 1b including longer study lists that should elicit stronger goal shifts. If providing JOLs shifts learners’ goals toward prioritizing easier over more difficult items (see changed-goal account), this should result in more restudy decisions for related pairs and/or less restudy decisions for unrelated pairs.

## Method

We report how we determined our sample sizes, all data exclusions, all manipulations, and all measures. All experiments follow the Journal Article Reporting Standard (JARS; Appelbaum et al., 2018). All experiments were conducted in line with international and local ethics guidelines; they were exempt from review by the local ethics committee. The materials, data, and analytical code of all experiments are available via the Open Science Framework (OSF) at https://osf.io/a53yw/. All experiments were preregistered and the preregistrations are available via the OSF – Experiment 1a: https://osf.io/h3ce9/; Experiment 1b: https://osf.io/q4a85/

### Design and participants

Experiments 1a and 1b used a ﻿2 (relatedness: related, unrelated) *×* 2 (judgment group: JOL, no JOL) mixed design with relatedness manipulated within participants and judgment group manipulated between participants.

#### Experiment 1a

An a priori power analysis using G*Power (Faul et al., 2007) indicated that 64 participants per group would be sufficient for detecting medium-sized effects (*d* = 0.50, e.g., see Soderstrom et al., 2015, Experiment 1b; also, see recommendations by Ingendahl et al., 2025) in two-tailed *t* tests for independent samples with α =.05 and (1 – β) =.80. Further, a sensitivity analysis showed that a total sample size of *N* = 128 participants allowed for detecting a moderately sized (*f* = 0.12) interaction between relatedness and judgment group in a mixed ANOVA.

To arrive at the preregistered sample size, we recruited 144 German students in exchange for partial course credit. We excluded three participants reporting technical malfunctions, one participant reporting deception (writing down word pairs during study), and no participants due to not providing JOLs in more than 20% of the study trials. The final sample comprised *N* = 140 participants that were randomly assigned to one of the judgment groups (*n* = 68 in the JOL group and *n* = 72 in the no-JOL group). All participants (102 women, 37 men, one diverse) were students at the Faculty of Human Sciences or the Faculty of Social Sciences, had a mean age of 22.59 years (*SD* = 3.49), and were mostly (92.86%) native in German.

#### Experiment 1b

To address the possibility that the sample size of Experiment 1a may have been too small to detect rather subtle differences in restudy decisions due to making JOLs, we aimed to achieve higher statistical power for detecting smaller effects in Experiment 1b. To do so, we planned to recruit a larger sample than in Experiment 1a and relied on using one-tailed tests (instead of two-tailed tests as in Experiment 1a) for testing directional preregistered hypotheses. Power analyses using G*Power (Faul et al., 2007) indicated that increasing the sample size from *N* = 128 to *N* = 200 (100 participants per judgment group) would be sufficient for detecting a small-to-moderate effect (*d* = 0.35) of judgment group in one-tailed *t* tests for independent samples (comparable to the size of JOL reactivity observed for word pairs in the meta-analysis by Ingendahl et al., 2025) and a small (*f* = 0.10) interaction between relatedness and judgment group in a mixed ANOVA with α =.05 and (1 – β) =.80.

To arrive at the preregistered sample size, we recruited 210 German students. 107 participants were recruited at two German universities (University sample), and 103 participants were recruited on Prolific (Prolific sample). Supplementing the University sample with participants recruited on Prolific was essential for obtaining a larger sample and increasing statistical power. Participants in the University sample were students at the Faculty of Human Sciences or the Faculty of Social Sciences and could choose to receive partial course credit or monetary compensation at a rate of 12.00€ per hour. Participants recruited on Prolific were high school or university students^1^ and received monetary compensation at a rate of £10.49 per hour (equivalent to 12.00€ per hour). We excluded two participants reporting technical malfunctions, three participants reporting deception (such as writing down word pairs during study), one participant above the age of 35 years, and one participant not fluent in German. No participants were excluded due to not providing JOLs in more than 20% of the study trials. The final sample comprised *N* = 202 participants that were randomly assigned to one of the judgment groups (*n* = 101 in each judgment group). Participants (105 women, 94 men, three diverse) had a mean age of 23.52 years (*SD* = 3.82) and were mostly native (85.64%) in German (see Online Supplementary Materials (OSM) for further demographic information by subsample and equivalence tests for age, gender, and language proficiency).

### Materials

#### Experiment 1a

Two lists of 20 German target words with one related and one unrelated German cue word each were taken from Melinger and Weber (2006) and used as materials to counterbalance assignment of target words to related and unrelated word pairs. Each list contained 20 strongly related word pairs (e.g., “guitar – music”) with a mean forward associative strength of .39 (*SD* =.09, range =.21–.59) and 20 unrelated word pairs (e.g., “bone – music”) with a mean forward associative strength of .00. Each participant was presented with all related word pairs from one list and all unrelated word pairs from the other list, with related and unrelated pairs from both lists presented equally often to participants in the JOL and no-JOL groups. Within and across lists, related and unrelated word pairs were equal in length (*M* = 5.47, *SD* = 0.99, range = 4.00–9.00), valence (*M* = 0.88, *SD* = 0.65, range = –0.66–2.15), arousal (*M* = 2.43, *SD* = 0.44, range = 1.44–3.47), frequency per million (*M* = 72.35, *SD* = 93.12, range = 1.75–409.25), and concreteness (*M* = 5.89, *SD* = 0.48, range = 4.66–6.67; normed values for valence, arousal, frequency, and concreteness were taken from Lahl et al., 2009). Four additional word pairs (two related, two unrelated) were used as buffer pairs presented at the beginning of the study phase and were not included in any analysis.

#### Experiment 1b

Two lists of 30 German target words with one related and one unrelated German cue word each were taken from Melinger and Weber (2006) and used as materials to counterbalance assignment of target words to related and unrelated word pairs. Each list contained 30 strongly related word pairs (e.g., “pillow – feather”) with a mean forward associative strength of .38 (*SD* =.10, range =.20–.59) and 30 unrelated word pairs (e.g., “cake – feather”) with a mean forward associative strength of .00. Each participant was presented with all related word pairs from one list and all unrelated word pairs from the other list, with related and unrelated pairs from both lists presented equally often to participants in the JOL and no-JOL groups. Within and across lists, related and unrelated word pairs were equal in length (*M* = 5.38, *SD* = 0.92, range = 3.00–9.00), valence (*M* = 0.82, *SD* = 0.67, range = –0.76–2.15), arousal (*M* = 2.43, *SD* = 0.50, range = 1.51–3.93), frequency per million (*M* = 58.20, *SD* = 76.01, range = 2.58–409.25), and concreteness (*M* = 5.83, *SD* = 0.46, range = 4.20–6.72; normed values for valence, arousal, frequency, and concreteness were taken from Lahl et al., 2009). Four additional word pairs (two related, two unrelated) were used as buffer pairs presented at the beginning of the study phase and were not included in any analysis.

### Procedure

After providing informed consent, participants were asked to study 44 word pairs (Experiment 1a) or 64 word pairs (Experiment 1b) for a later memory test in which they would have to recall the second word of each word pair when being presented with the first word of each pair. Participants then studied the word pairs for 6 s each. Whereas participants in the no-JOL group studied the word pairs for the full study time, participants in the JOL group were prompted after 2 s to provide a JOL (“probability of coming up with the second word when presented with the first word at the later test?”) by dragging a bar along a continuous 0–100 horizontal scale within the remaining 4 s. If participants in the JOL group failed to provide a JOL within this time, the word pair was replaced by the prompt “Please give your judgment for the next pair within the provided time” that remained on screen for 3 s.

Immediately after studying each word pair, all participants were asked to provide a self-paced restudy decision by indicating whether they would like to restudy the respective word pair if they had the opportunity (“Would you like to study this word pair again?” – “yes” or “no”). Prior to studying, participants were informed that they would be asked to make restudy decisions but that they would not have the opportunity to restudy the selected word pairs as part of the study: “Immediately after studying each word pair, we will ask you to indicate whether you would like to study the word pair again in order to perform well on the later memory test. If you would like to study the word pair again, please select *yes*. If you would not like to study the word pair again, please select *no*. Unfortunately, we will not be able to give you the opportunity to restudy the selected word pairs. Nevertheless, we are interested in which word pairs you would choose to study again if you had the opportunity.” After providing a restudy decision, participants proceeded to studying the next word pair.

After studying all word pairs, participants solved visual reasoning items (Chierchia et al., 2019) for 3 min, and then completed a self-paced cued-recall test. In the test, participants were presented with the cue word of each pair and had to type in the respective target word. Word pairs were presented in new random orders at study and test.

### Analytical approach

*R* (Version 4.3.3; R Core Team, 2024) and *R Studio* (Version 2026.1.0.5392; Posit team, 2026) were used for data preparation and all analyses reported in this study. We used *afex* (Version 1.3-1; Singmann et al., 2024) for all reported ANOVAs, *effsize* (Version 0.8.1; Torchiano, 2016) for calculating effect sizes for *t* tests, and *lme4* (Version 1.1–35.3; Bates et al., 2015) and *lmerTest* (Version 3.1–3; Kuznetsova et al., 2017) for all frequentist multilevel analyses, and the *brms* package (Version 2.22.0, Bürkner, 2017) for all Bayesian multilevel analyses. All reported analyses pertaining to the individual experiments were preregistered. Integrative data analyses across Experiments 1a and 1b were not preregistered.

Word pairs were coded as correctly recalled if the response matched the target word that had been studied with the presented cue word, including minor spelling errors or pluralization. For restudy decisions, we excluded trials in the JOL group for which participants failed to provide a JOL within the study duration because the reminder to provide a JOL increased the delay between studying the word pair and providing a restudy decision. This resulted in an exclusion of 93 (1.67%) restudy decisions in Experiment 1a (related: 42 or 1.50%; unrelated: 51 or 1.82%) and 166 (1.37%) restudy decisions in Experiment 1b (related: 87 or 1.44%; unrelated: 79 or 1.30%).

Preliminary exploratory analyses (see OSM) showed that the assignment of lists to related and unrelated word pairs did not interact with judgment group on cued-recall performance, Experiment 1a: *F*s ≤ 3.20, *p*s ≥.076, ${\eta}_{p}^{2}$ s ≤.02; Experiment 1b: *F*s ≤ 1.01, *p*s ≥.316, ${\eta}_{p}^{2}$ s ≤.01, or restudy decisions, Experiment 1a: *F*s ≤ 0.75, *p*s ≥.389, ${\eta}_{p}^{2}$ s ≤.01; Experiment 1b; *F*s ≤ 2.18, *p*s ≥.141, ${\eta}_{p}^{2}$ s ≤.01. Further exploratory analyses showed that effects on cued-recall performance, *F*s ≤ 0.51, *p*s ≥.447, ${\eta}_{p}^{2}$ s <.01, and restudy decisions, *F*s ≤ 1.78, *p*s ≥.184, ${\eta}_{p}^{2}$ s ≤.01, did not differ by subsample (University sample or Prolific sample) in Experiment 1b (see OSM). Therefore, data were collapsed across list assignments and subsamples in all analyses reported below.

For each experiment and as a manipulation check, we analyzed JOLs with a paired *t* test comparing JOLs for related and unrelated word pairs. We analyzed cued-recall performance and restudy decisions (percentage of pairs selected for restudy) with 2 (relatedness: related, unrelated) *×* 2 (judgment group: JOL, no JOL) mixed ANOVAs and followed up on significant interactions with paired *t* tests comparing differences between related and unrelated word pairs separately in the JOL and no-JOL groups (relatedness effect) and with two-sample *t* tests comparing differences between judgment groups separately for related and unrelated word pairs (reactivity effect). As preregistered, we tested directional hypotheses using two-tailed *t* tests in Experiment 1a but, to increase statistical power, using one-tailed *t* tests in Experiment 1b. The conclusions derived from one-tailed and two-tailed statistical tests were identical for all measures. For transparency, we annotate all one-tailed* t* tests.

To synthesize trends across individual experiments and ensure even higher statistical power for hypothesis tests, we conducted exploratory analyses integrating data across Experiments 1a and 1b. We relied on mixed models for synthesizing effects across experiments to account for differences between experiments and sample populations, as well as overlap and differences in study materials. For JOLs, we relied on a linear mixed model to test the effect of relatedness across experiments. For cued recall and restudy decisions, we used generalized mixed models to test whether each measure differed by relatedness and judgment group. We included random intercepts for participants and word pairs to account for between-cluster variance and random slopes for relatedness nested within participants in all models.^2^ Relatedness (–0.5 = unrelated pair, 0.5 = related pair) and judgment group (–0.5 = no JOL, 0.5 = JOL; cued recall and restudy decisions only) were entered as effect-coded predictors into the analysis. To control for differences between experiments, all models included an effect-coded predictor for the main effect of experiment (–0.5 = Experiment 1a, 0.5 = Experiment 1b). To more thoroughly evaluate the evidence for our conclusions, we ran equivalent Bayesian mixed models for JOLs, cued recall, and restudy decisions. We report Bayes factors and 95% credibility intervals for parameter estimates for ambiguous non-significant frequentist effects. Bayesian mixed models were based on weakly informative priors following recommendations by Gelman and colleagues (2008) for prior specifications in mixed-effects regression models. We relied on weakly informative priors to regularize extreme and implausible parameter values, while still allowing Bayes factors to be estimated via model comparison. We avoided specifying strongly informed priors to prevent biasing the findings toward any specific parameter values because the integrative analyses were explorative in nature and no strong theory-driven expectations for effect sizes were available. Full details on prior specifications and results of all Bayesian mixed models are reported in the OSM.

The results we present focus on the integrative data analyses across Experiments 1a and 1b to ensure greater robustness and conclusiveness of findings. For completeness, we additionally present results of the preregistered analyses for each experiment. The α level was set to.05 for all frequentist analyses. We interpret Bayes factors based on the following conventions: <1/10 = strong evidence for H_0_; 1/10–1/3 = moderate evidence for H_0_; 1/3–1 = anecdotal evidence for H_0_; 1–3 = anecdotal evidence for H_1_; 3–10 = moderate evidence for H_1_; >10 = strong evidence for H_1_ (van Doorn et al., 2021).

## Results

### JOLs

The linear mixed model (Table 1) revealed higher JOLs for related than unrelated pairs, *b* = 40.52, *p* <.001. Analyses of each experiment replicated this finding, Experiment 1a: *t*(67) = 17.55, *p* <.001, *d* = 2.04, *M*_*related*_ = 65.70, *SD*_*related*_ = 17.95, *M*_*unrelated*_ = 29.94, *SD*_*unrelated*_ = 17.00; Experiment 1b: *t*(100) = 25.58, *p* <.001 (one-tailed), *d* = 2.89, *M*_*related*_ = 69.44, *SD*_*related*_ = 16.36, *M*_*unrelated*_ = 25.28, *SD*_*unrelated*_ = 13.99.

**Table 1:** Linear mixed model for prediction of judgments of learning (JOLS) across Experiments 1a and 1b

Predictor	*b*	95% CI	*p*
(Intercept)	47.47	[45.19, 49.75]	<.001
Experiment	–0.83	[–5.19, 3.53]	.709
Relatedness	**40.52**	**[37.40, 43.65]**	**<.001**
Number of observations	8,521		
Number of participants	169		
Number of word pairs^a^	163		
Conditional/Marginal *R*^*2*^	.56/.45		

Experiment (–0.5 = Experiment 1a, 0.5 = Experiment 1b), Judgment group (–0.5 = no JOL, 0.5 = JOL), and Relatedness (–0.5 = unrelated pair, 0.5 = related pair) were entered as effect-coded predictors. *95% CI* refers to the frequentist confidence interval. *Conditional R*^*2*^ refers to the variance explained by random and fixed effects, whereas *Marginal R*^*2*^ is the variance explained by fixed effects only

^a^ *Number of word pairs* refers to the number of unique word pairs studied across Experiments 1a and 1b (100 unrelated, 63 related due to overlap across experiments)

Bold values indicate significant predictors at *p* <.05

### Cued recall

**Fig. 1:**
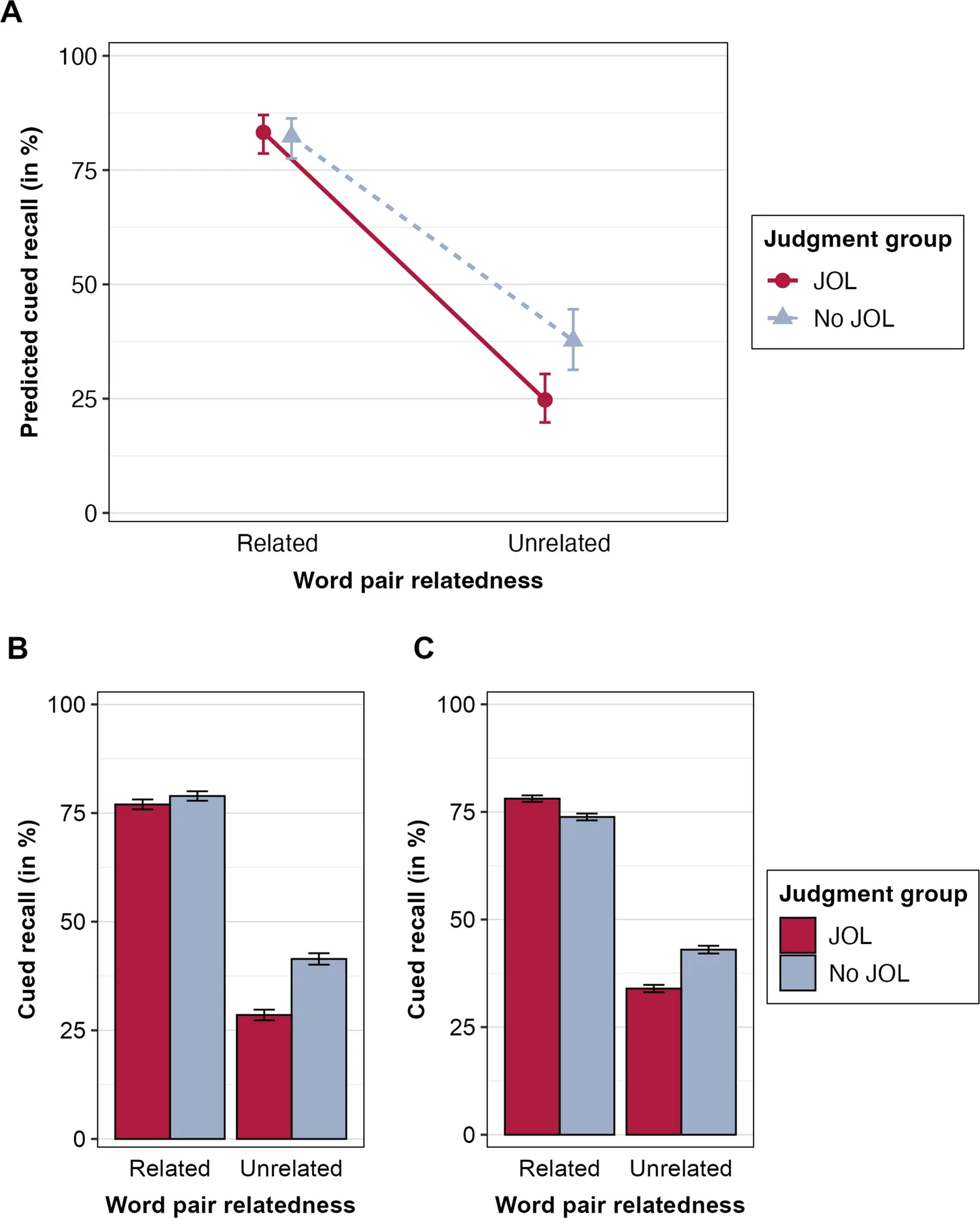
Predicted probabilities of cued recall across experiments (**A**) and mean percentage of cued recall in Experiments 1a (**B**) and 1b (**C**). Error bars represent 95% confidence intervals for regression weights of the frequentist generalized mixed model in Panel A and one standard error of the mean in Panels B and C

Figure 1 presents cued recall. Generalized mixed models showed higher recall for related than unrelated pairs, *b* = 2.38, *p* <.001 (Table 2A) and that this differed by judgment group (Fig. 1A), *b* = 0.68, *p* <.001. Follow-up analyses revealed no reactivity for related pairs, *b* = 0.07, *p* =.667 (see Table 2B), but negative reactivity for unrelated pairs,* b* = –0.64, *p* <.001 (Table 2C).

**Table 2:** Generalized mixed model for prediction of cued recall across Experiments 1a and 1b

Predictor	(A) Related and unrelated pairs			(B) Related pairs			(C) Unrelated pairs		
	*b*	95% CI	*p*	*b*	95% CI	*p*	*B*	95% CI	*p*
(Intercept)	0.40	[0.20, 0.60]	<.001	1.60	[1.36, 1.84]	<.001	–0.82	[–1.07, –0.57]	<.001
Experiment	–0.09	[–0.40, 0.22]	.569	–0.16	[–0.47, 0.16]	.322	0.20	[–0.29, 0.70]	.422
Judgment group	–0.27	[–0.56, 0.01]	.061	0.07	[–0.23, 0.37]	.667	**–0.64**	**[–0.96, –0.32]**	**<.001**
Relatedness	**2.38**	**[2.08, 2.68]**	**<.001**						
Judgment group × Relatedness	**0.68**	**[0.44, 0.91]**	**<.001**						
Number of observations	17,640			8,820			8,820		
Number of participants	340			340			340		
Number of word pairs^a^	163			63			100		
Conditional/Marginal *R*^*2*^	.55/.20			.40/<.01			.47/.02		

Experiment (–0.5 = Experiment 1a, 0.5 = Experiment 1b), Judgment group (–0.5 = no JOL, 0.5 = JOL), and Relatedness (–0.5 = unrelated pair, 0.5 = related pair) were entered as effect-coded predictors. *95% CI* refers to the frequentist confidence interval. *Conditional R*^*2*^ refers to the variance explained by random and fixed effects, whereas *Marginal R*^*2*^ is the variance explained by fixed effects only

^a^ *Number of word pairs* refers to the number of unique word pairs studied across Experiments 1a and 1b (100 unrelated, 63 related due to overlap across experiments)

Bold values indicate significant predictors at *p* <.05

Analyses in each experiment (Figs. 1B and 1 C) were consistent with these results. ANOVAs (Table 3A) indicated a significant effect of relatedness revealing higher recall for related than unrelated pairs and a significant interaction showing a larger relatedness effect in the JOL than the no-JOL group; Experiment 1a, JOL group: *t*(67) = 20.66, *p* <.001, *d* = 2.45; no-JOL group: *t*(71) = 16.47, *p* <.001, *d* = 1.68; Experiment 1b, JOL group: *t*(100) = 24.41, *p* <.001 (one-tailed), *d* = 2.13; no-JOL group: *t*(100) = 17.56, *p* <.001 (one-tailed), *d* = 1.23. This was due to no reactivity for related pairs, Experiment 1a: *t*(138) = –0.40, *p* =.690, *d* = –0.07; Experiment 1b: *t*(200) = 1.51, *p* =.066 (one-tailed), *d* = 0.21, but negative reactivity for unrelated pairs, Experiment 1a: *t*(138) = –3.12, *p* =.002, *d* = –0.52; Experiment 1b: *t*(200) = –2.61, *p* =.005 (one-tailed), *d* = –0.37.

**Table 3:** Mixed ANOVAs with the factors relatedness and judgment group for cued-recall performance and restudy decisions

Experiment and effect	(A) Cued-recall performance				(B) Restudy decisions			
	*F*	*df*	*p*	${\eta}_{p}^{2}$	*F*	*df*	*p*	${\eta}_{p}^{2}$
Experiment 1a								
Relatedness	690.22	1, 138	<.001	.83	166.89	1, 138	<.001	.55
Judgment group	4.27	1, 138	.041	.03	0.26	1, 138	.612	<.01
Relatedness × Judgment group	10.13	1, 138	.002	.07	3.84	1, 138	.052	.03
Experiment 1b								
Relatedness	886.66	1, 200	<.001	.82	165.77	1, 200	<.001	.45
Judgment group	0.68	1, 200	.409	<.01	0.00	1, 200	.961	<.01
Relatedness × Judgment group	27.92	1, 200	<.001	.12	3.33	1, 200	.069	.02

Relatedness was manipulated within participants, and Judgment group was manipulated between participants

### Restudy decisions

**Fig. 2:**
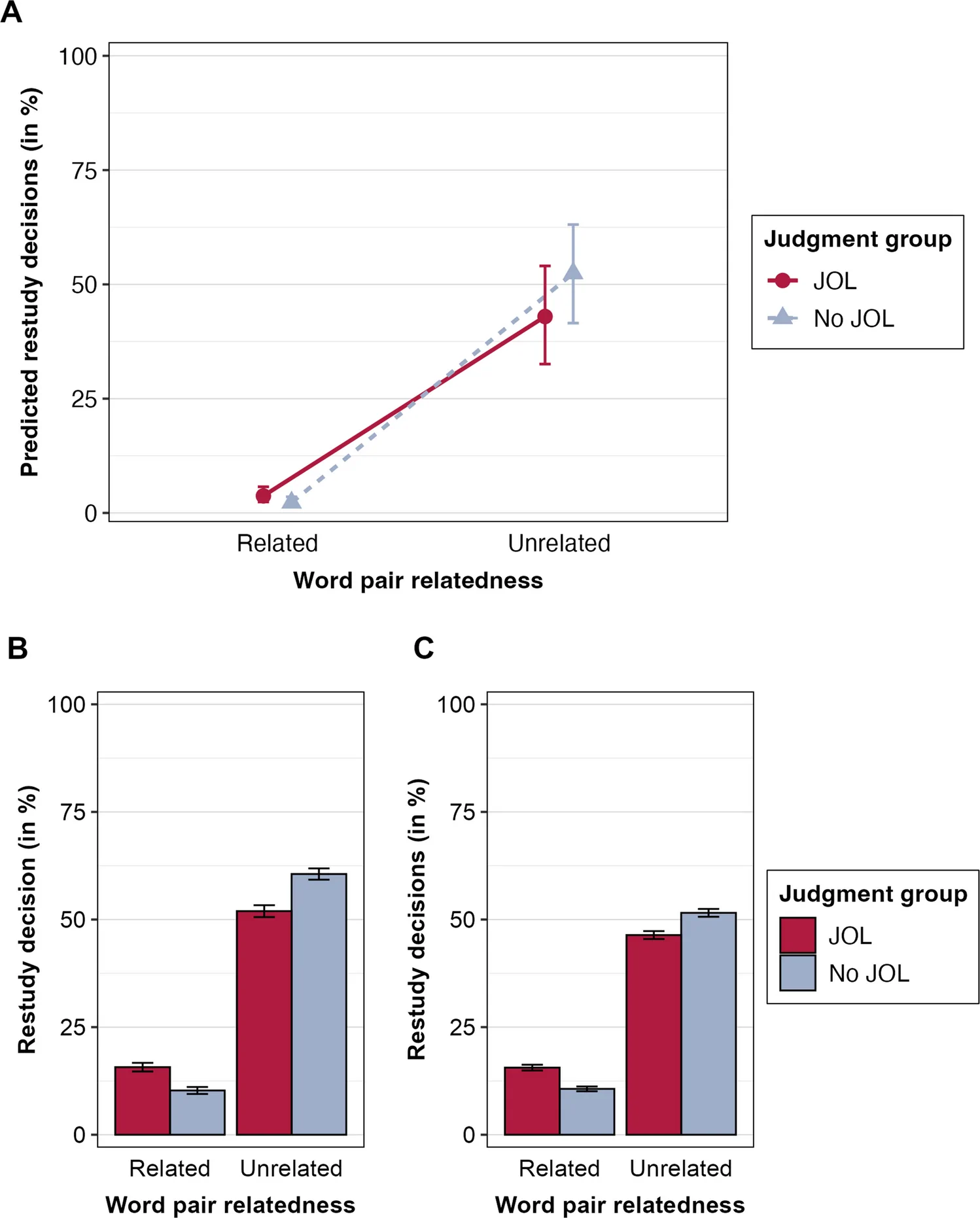
Predicted probabilities of restudy decisions across Experiments (**A**) and mean percentage of restudy decisions in Experiments 1a (**B**) and 1b (**C**). Error bars represent 95% confidence intervals for regression weights of the frequentist generalized mixed model in Panel A and one standard error of the mean in Panels B and C

Figure 2 presents restudy decisions. Generalized mixed models revealed that participants chose related pairs less frequently for restudy than unrelated pairs, *b* = –3.42, *p* <.001 (Table 4A) and that this effect differed by judgment group, *b* = 0.90, *p* =.011. The numerical values presented in Fig. 2 may give the impression that the JOL group chose related pairs more frequently and unrelated pairs less frequently for restudy than the no-JOL group (as predicted by the changed-goal account), but this was not supported by statistical analyses: Groups did not differ significantly in restudy decisions for related pairs, *b* = 0.57, *p* =.060, or unrelated pairs, *b* = –0.36, *p* =.232, and judgment group and experiment explained virtually no variance in restudy decisions for related or unrelated pairs, *marginal R*^*2*^s =.01. Aligning with frequentist inference, Bayesian inference (Table S4, OSM) supported that the effect of relatedness interacted with judgment group, Bayesian 95% CI [0.17, 1.56], *BF*_*10*_ = 18.76, but provided no evidence for an effect of judgment group on restudy decisions for related pairs, Bayesian 95% CI [–0.06, 1.14], *BF*_*10*_ = 0.46, or unrelated pairs, Bayesian 95% CI [–0.96, 0.27], *BF*_*10*_ = 0.22.

**Table 4:** Generalized mixed model for prediction of restudy decisions across Experiments 1a and 1b

Predictor	(A) Related and unrelated pairs			(B) Related pairs			(C) Unrelated pairs		
	*b*	95% CI	*p*	*b*	95% CI	*p*	*b*	95% CI	*p*
(Intercept)	–1.74	[–2.03, –1.46]	<.001	–3.40	[–3.77, –3.03]	<.001	0.01	[–0.31, 0.32]	.964
Experiment	–0.31	[–0.84, 0.22]	.255	–0.03	[–0.65, 0.58]	.917	–0.62	[–1.25, 0.01]	.052
Judgment group	0.07	[–0.44, 0.58]	.793	0.57	[–0.03, 1.17]	.060	–0.36	[–0.96, 0.23]	.232
Relatedness	**–3.42**	**[–3.83, –3.00]**	**<.001**						
Judgment group × Relatedness	**0.90**	**[0.21, 1.59]**	**.011**						
Number of observations	17,381			8,691			8,690		
Number of participants	340			340			340		
Number of word pairs^a^	163			63			100		
Conditional/Marginal *R*^*2*^	.76/.22			.65/.01			.70/.01		

Experiment (–0.5 = Experiment 1a, 0.5 = Experiment 1b), Judgment group (–0.5 = no JOL, 0.5 = JOL), and Relatedness (–0.5 = unrelated pair, 0.5 = related pair) were entered as effect-coded predictors. *95% CI* refers to the frequentist confidence interval. *Conditional R*^*2*^ refers to the variance explained by random and fixed effects, whereas *Marginal R*^*2*^ is the variance explained by fixed effects only

^a^ *Number of word pairs* refers to the number of unique word pairs studied across Experiments 1a and 1b (100 unrelated, 63 related due to overlap across experiments)

Bold values indicate significant predictors at *p* <.05

Analyses in each experiment (Figs. 2B and 2 C) were overall consistent with these results. ANOVAs (Table 3A) indicated a significant relatedness effect revealing that participants chose to restudy related pairs less frequently than unrelated pairs. Critically, the interaction was not significant, providing no reliable evidence for a difference in the relatedness effect between groups, Experiment 1a, JOL group: *t*(67) = 7.54, *p* <.001, *d* = 1.12; no-JOL group: *t*(71) = 10.81, *p* <.001, *d* = 1.75; Experiment 1b, JOL group: *t*(100) = 7.12, *p* <.001 (one-tailed), *d* = 0.96; no-JOL group: *t*(100) = 11.21, *p* <.001 (one-tailed), *d* = 1.44. There was no significant reactivity for related pairs, Experiment 1a: *t*(138) = 1.20, *p* =.232, *d* = 0.20; Experiment 1b: *t*(200) = 1.56, *p* =.060 (one-tailed), *d* = 0.22, or unrelated pairs, Experiment 1a: *t*(138) = –1.39, *p* =.168, *d* = –0.23; Experiment 1b, *t*(200) = –1.04, *p* =.150 (one-tailed), *d* = –0.15.

## General discussion

The present study examined whether soliciting JOLs alters metacognitive control, as indicated by restudy decisions. Across two experiments, we observed no memory reactivity for related pairs but negative memory reactivity for unrelated pairs. Critically, we observed no reliable change in restudy decisions for related or unrelated pairs due to soliciting JOLs. Our findings suggest that negative reactivity for unrelated pairs may not be explained by changes in metacognitive control decisions during learning.

These experiments have theoretical implications for JOL reactivity because they test a core assumption of the *changed-goal account* (Mitchum et al., 2016), proposing that JOLs result in prioritizing easier items at the expense of more difficult items. If this assumption holds, the JOL group should exhibit an increase in restudy decisions for related pairs and/or a decrease for unrelated pairs compared to the no-JOL group. While making JOLs consistently resulted in no reactivity for related pairs and negative reactivity for unrelated pairs in recall, restudy decisions did not differ reliably by the requirement to provide JOLs. Integrative analyses across experiments supported this. Despite showing a significant interaction between relatedness and group in the direction predicted by the changed-goal account, integrative analyses revealed no group differences in restudy decisions for related or unrelated pairs with Bayesian inference indicating no evidence for a JOL reactivity effect and judgment group explaining virtually no variance in restudy decisions (related: $\Delta {R}^{2}$ = 0.90%, unrelated: $\Delta {R}^{2}$ = 0.30%).

The observation that JOLs impaired recall for unrelated pairs but did not alter restudy decisions is inconsistent with the changed-goal account. If JOLs impair memory for unrelated pairs through deprioritization of items perceived as more difficult, the JOL group should select unrelated pairs less frequently for restudy than the no-JOL group. Thus, the present findings speak against a conceptualization of this account in which goal changes manifest as general changes in metacognitive control decisions. They do not rule out other conceptualizations, including those in which goal changes manifest in specific learning strategy changes (e.g., Double et al., 2026; Ingendahl & Undorf, 2026a).

The present findings reveal no clear differences in restudy decisions for related pairs when soliciting JOLs. Because we observed no memory reactivity for related pairs, we cannot exclude that JOLs may alter restudy decisions when positive reactivity for related pairs occurs. Nevertheless, there are good reasons to question whether the changed-goal account can explain JOL reactivity in restudy decisions (and memory) for related pairs, even when positive memory reactivity is present. First, the mechanism proposed by the changed-goal account for positive and negative reactivity would then apply to related pairs only. Second, recent empirical work favors alternative explanations of positive reactivity over the changed-goal account (e.g., Li et al., 2024; Maxwell & Huff, 2023).

The present findings also have implications for other JOL reactivity accounts. The lack of JOL-associated differences in restudy decisions is consistent with the *cue-strengthening account* (Soderstrom et al., 2015) attributing reactivity to strengthened processing of cues (e.g., relatedness) utilized for JOLs. As cue strengthening may occur rather automatically (Maxwell & Huff, 2024), it could operate independently of control. However, because the cue-strengthening account predicts positive memory reactivity, it is inconsistent with this study.^3^ The present findings are consistent with the *dual-task account* (Mitchum et al., 2016) of negative reactivity, proposing that JOLs interfere with learning and thus impair memory, particularly when learning is demanding (e.g., for unrelated pairs). The dual-task account aligns with no memory reactivity for related pairs and negative reactivity for unrelated pairs. Because the dual-task account focuses on interference during learning, it does not predict any effects of JOLs on restudy decisions and is thus consistent with the present findings.

The present study comes with limitations from examining non-consequential restudy decisions during learning and manipulating relatedness. Participants selected pairs for restudy during learning without completing a restudy phase, because restudying, particularly of selected items, could have exaggerated or obscured JOL reactivity on memory, thereby precluding an examination of restudy decisions in context of JOL reactivity. Requesting non-consequential restudy decisions and informing participants about this might have resulted in less pronounced goal changes, attenuating JOL-associated effects. At the same time, consequential restudy decisions could have introduced motivational influences unrelated to goal changes (Dunlosky & Ariel, 2011), complicating conclusions about reactivity on restudy decisions. For example, participants might have selected fewer unrelated pairs for restudy to reduce the demands of restudying (see Schulz et al., 2026), irrespective of providing JOLs. Importantly, non-consequential restudy decisions have been used in prior studies (e.g., Luna et al., 2019; Metcalfe & Finn, 2008; Soderstrom & Rhodes, 2014; Tauber & Rhodes, 2012), and Hanczakowski et al. (2014) observed identical patterns for consequential and non-consequential restudy decisions. Similarly, while assessing restudy decisions during learning immediately after JOLs might have affected the relationship between JOLs and restudy decisions or restricted the strength of observable changes, Schaper and Bayen (2021) did not observe differences in restudy decisions assessed during or after learning. Further, the replication of the often-observed preference for restudying difficult items (e.g., Shanks & Serra, 2014; Thiede & Dunlosky, 1999) in our experiments supports the validity of the restudy decisions assessed in this study as a measure of metacognitive control. Another limitation concerns the use of relatedness to manipulate perceived item difficulty. While this approach is standard in JOL reactivity research (see Ingendahl et al., 2025), the strong effect of relatedness on perceived difficulty and its categorical manipulation may have obscured more subtle changes in metacognitive control. Future research using manipulations with weaker effects on perceived difficulty (e.g., concreteness) or manipulating item difficulty along a continuum (e.g., Undorf et al., 2018) may provide a more sensitive test of JOL reactivity in metacognitive control.

In conclusion, this study demonstrates that soliciting JOLs impairs memory for unrelated pairs but does not reliably alter restudy decisions for these pairs. It thus questions the adequacy of the changed-goal account for explaining negative JOL reactivity and points toward the need for future research on reactivity of JOLs to metacognitive control across various conditions.

## Supplementary Information

Below is the link to the electronic supplementary material.

Supplementary file1 (PDF 200 KB)

## Data Availability

All data and materials of the reported experiments are available via the Open Science Framework and can be accessed at https://osf.io/a53yw/.

## Footnotes

1 To recruit participants of similar educational background and age in the University and Prolific samples, we restricted recruitment to students using Prolific’s filtering system. Although most German students on Prolific are University students from various disciplines, the population also includes high school students in their final years who are at least 18 years old.

2 We did not include random intercepts for experiments as this resulted in singular fit due to lack of variance.

3 Note that the cue-strengthening account can explain the observed memory reactivity when supplemented with the dual-task account (as suggested by Janes et al., 2018, and Undorf et al., 2024), in which positive effects of cue strengthening are counteracted by dual-task costs from making JOLs. Given the high dual-task costs expected under short experimenter-paced study durations as in the present study, the two accounts together explain the no reactivity for related pairs and negative reactivity for unrelated pairs observed in this study.

## Code availability

All analytical code underlying all reported results is available via the Open Science Framework and can be accessed at https://osf.io/a53yw/.

## Declarations

### Open practices statement

All experiments reported in this research were preregistered and all presented analyses were preregistered unless stated otherwise. The preregistrations are available via the Open Science Framework.

*Experiment 1a*: https://osf.io/h3ce9/

*Experiment 1b*: https://osf.io/q4a85/

### Author Note

All data, materials, and analytic code are available on the Open Science Framework and can be accessed at https://osf.io/a53yw/. The authors thank Jula Remiorz for her help in preparing materials for Experiment 1a.
