# Supplementary material for: Are restudy decisions reactively altered by judgments of learning? Insights from related and unrelated word pairs
Source: Psychon Bull Rev. 2026 Oct 22;33(8):261. doi: 10.3758/s13423-026-03030-2 (PMC13690918; doi:10.3758/s13423-026-03030-2)
Supplement: Supplementary file 1 [file 13423_2026_3030_MOESM1_ESM.pdf]

Supplementary Materials

Experiment 1a

Counterbalancing of List Assignment

JOLs. We conducted an exploratory 2 (relatedness: related, unrelated) × 2 (list: A, B) mixed ANOVA on JOLs with relatedness as within-subjects factor and list as between-subjects factor. The main effect of list was not significant, $F(1, 66) = 0.58$, $p = .449$, $\eta_p^2 = .01$. A significant interaction, $F(1, 66) = 9.85$, $p = .003$, $\eta_p^2 = .13$, indicated that the size of the relatedness effect on JOLs was smaller in List A, $t(34) = 10.62$, $p < .001$, $d = 1.63$, than in List B, $t(32) = 16.27$, $p < .001$, $d = 2.60$.

Cued Recall. We conducted an exploratory 2 (relatedness: related, unrelated) × 2 (judgment group: JOL, no JOL) × 2 (list: A, B) mixed ANOVA on cued-recall performance with relatedness as within-subjects factor and judgment group and list as between-subjects factors. A significant main effect of list indicated that cued recall was lower in List A than in List B, $F(1, 136) = 12.96$, $p < .001$, $\eta_p^2 = .09$, $M_A = .51$, $SD_A = .32$, $M_B = .62$, $SD_B = .28$. A significant interaction between relatedness and list, $F(1, 136) = 9.85$, $p = .003$, $\eta_p^2 = .13$, indicated that the size of the relatedness effect on cued recall was larger in List A, $t(70) = 20.29$, $p < .001$, $d = 2.16$, than in List B, $t(68) = 16.23$, $p < .001$, $d = 1.89$. No other effects including list were significant: Judgment group × List, $F(1, 136) = 0.47$, $p = .493$, $\eta_p^2 < .01$; Relatedness × Judgment group × List, $F(1, 136) = 3.20$, $p = .076$, $\eta_p^2 = .02$.

Restudy Decisions. We conducted an exploratory 2 (relatedness: related, unrelated) × 2 (judgment group: JOL, no JOL) × 2 (list: A, B) mixed ANOVA on restudy decisions with relatedness as within-subjects factor and judgment group and list as between-subjects factors. No main effects or interactions including list were significant: List, $F(1, 136) = 0.41$, $p = .523$, $\eta_p^2 < .01$; Relatedness × List, $F(1, 136) = 0.64$, $p = .424$, $\eta_p^2 = .01$; Judgment group × List,

$F(1, 136) = 0.30$, $p = .584$, $\eta_p^2 < .01$; Relatedness × Judgment group × List, $F(1, 136) = 0.75$, $p = .389$, $\eta_p^2 = .01$.

Experiment 1b

Supplementary Demographic Information

Table S1 presents demographic information for participants in Experiment 1b depending on the subsample. Equivalence tests for demographic information comparing the University sample and the Prolific sample revealed that the University sample was on average two years younger than the Prolific sample, $t(200) = 2.58$, $p < .001$, $d = 0.64$, and that the gender distribution differed between the subsamples, $\chi^2 = 21.58$, $p < .001$, with the University sample consisting of more female and fewer male participants than the Prolific sample. German language proficiency was equivalent across subsamples, $\chi^2 = 1.13$, $p = .326$. The observed differences in age and gender between the subsamples are not directly relevant to the present study's research question, because none of the primary dependent variables differed by subsample (see section Subgroup Analysis by Sample below).

Table S1

Demographic Information by Sample in Experiment 1b

	University sample	Prolific sample
N	102	100
n_{JOL}	51	50
$n_{No\ JOL}$	51	50
Age	22.36 (2.92)	24.71 (4.26)
Gender		
Female	69	36
Male	31	63
Diverse	2	1
German Language proficiency		
Native	90	83
Fluent	12	17

Note. Values in parentheses present one standard deviation.

Counterbalancing of List Assignment

JOLs. We conducted an exploratory 2 (relatedness: related, unrelated) × 2 (list: A, B) mixed ANOVA on JOLs with relatedness as within-subjects factor and list as between-subjects factor. No main effects or interaction including list were significant: List, $F(1, 99) = 0.02$, $p = .903$, $\eta_p^2 < .01$; Relatedness × List, $F(1, 99) = 2.31$, $p = .132$, $\eta_p^2 = .02$.

Cued Recall. We conducted an exploratory 2 (relatedness: related, unrelated) × 2 (judgment group: JOL, no JOL) × 2 (list: A, B) mixed ANOVA on cued-recall performance with relatedness as within-subjects factor and judgment group and list as between-subjects factors. No main effects or interactions including list were significant: List, $F(1, 198) = 1.01$, $p = .316$, $\eta_p^2 = .01$; Relatedness × List, $F(1, 198) = 0.06$, $p = .809$, $\eta_p^2 < .01$; Judgment group × List, $F(1, 198) = 0.52$, $p = .472$, $\eta_p^2 < .01$; Relatedness × Judgment group × List, $F(1, 198) = 0.86$, $p = .354$, $\eta_p^2 < .01$.

Restudy Decisions. We conducted an exploratory 2 (relatedness: related, unrelated) × 2 (judgment group: JOL, no JOL) × 2 (list: A, B) mixed ANOVA on restudy decisions with relatedness as within-subjects factor and judgment group and list as between-subjects factors. No main effects or interactions including list were significant: List, $F(1, 198) = 2.18$, $p = .141$, $\eta_p^2 = .01$; Relatedness × List, $F(1, 198) = 1.38$, $p = .242$, $\eta_p^2 = .01$; Judgment group × List, $F(1, 198) = 2.05$, $p = .154$, $\eta_p^2 = .01$; Relatedness × Judgment group × List, $F(1, 198) = 0.41$, $p = .520$, $\eta_p^2 < .01$.

Subgroup Analysis by Sample

JOLs. We conducted an exploratory 2 (relatedness: related, unrelated) × 2 (subgroup: University sample, Prolific sample) mixed ANOVA on JOLs with relatedness as within-subjects factor and subgroup as between-subjects factor. No main effects or interaction

including subgroup were significant: Subgroup, $F(1, 99) = 0.02$, $p = .899$, $\eta_p^2 < .01$; Relatedness × Subgroup, $F(1, 99) = 0.15$, $p = .698$, $\eta_p^2 < .01$.

Cued Recall. We conducted an exploratory 2 (relatedness: related, unrelated) × 2 (judgment group: JOL, no JOL) × 2 (subgroup: University sample, Prolific sample) mixed ANOVA on cued-recall performance with relatedness as within-subjects factor and judgment group and subgroup as between-subjects factors. No main effects or interactions including subgroup were significant: Subgroup, $F(1, 198) = 0.35$, $p = .554$, $\eta_p^2 < .01$; Relatedness × Subgroup, $F(1, 198) = 0.51$, $p = .447$, $\eta_p^2 < .01$; Judgment group × Subgroup, $F(1, 198) = 0.10$, $p = .758$, $\eta_p^2 < .01$; Relatedness × Judgment group × Subgroup, $F(1, 198) = 0.60$, $p = .441$, $\eta_p^2 < .01$.

Restudy Decisions. We conducted an exploratory 2 (relatedness: related, unrelated) × 2 (judgment group: JOL, no JOL) × 2 (subgroup: University sample, Prolific sample) mixed ANOVA on restudy decisions with relatedness as within-subjects factor and judgment group and subgroup as between-subjects factors. No main effects or interactions including subgroup were significant: Subgroup, $F(1, 198) = 0.00$, $p = .967$, $\eta_p^2 < .01$; Relatedness × Subgroup, $F(1, 198) = 1.78$, $p = .184$, $\eta_p^2 = .01$; Judgment group × Subgroup, $F(1, 198) = 0.06$, $p = .806$, $\eta_p^2 < .01$; Relatedness × Judgment group × Subgroup, $F(1, 198) = 0.89$, $p = .347$, $\eta_p^2 < .01$.

Bayesian Integrative Data Analysis

To gain additional insights into a potential contribution of JOLs to restudy decisions, we repeated the exploratory integrative analysis using a Bayesian approach. We relied on the *brms* package (Version 2.22.0, Bürkner, 2017) for all Bayesian mixed models reported below. For JOLs, we used a linear mixed model to test the effect of relatedness on JOLs across both experiments. For cued recall and restudy decisions, we used generalized mixed models to test whether recall and restudy decisions, respectively, differed by relatedness and judgment group. All models included random intercepts for participants and word pairs to account for

between-cluster variance and random slopes for relatedness nested within. Relatedness (–0.5 = unrelated pair, 0.5 = related pair) and judgment group (–0.5 = no JOL, 0.5 = JOL; cued recall and restudy decisions only) were entered as effect-coded predictors into the analysis. To control for differences between experiments, all experiments included an effect-coded predictor for the main effect of experiment (–0.5 = Experiment 1a, 0.5 = Experiment 1b).

We estimated the models using four parallel Markov chains with 8,000 iterations per chain, including 2,000 warm-up iterations. In all models, we relied on weakly informative priors following recommendations by Gelman and colleagues (2008). For linear mixed effects models for JOLs, priors were defined as follows:

Fixed effects for all non-intercept coefficients followed a Student-*t* distribution with 3 degrees of freedom, mean 0, and scale 2.5, that is,

$$\beta_j \sim t_3(0, 2.5) \ .$$

The intercept followed a wider Student-*t* distribution with 3 degrees of freedom, mean 0, and scale 10, that is,

$$\beta_0 \sim t_3(0, 10) \ .$$

Standard deviations of the random effects followed a half-Student-*t* distribution with 3 degrees of freedom, mean 0, and scale 2.5, that is,

$$\sigma_k \sim t_3^+(0, 2.5),$$

and the residual standard deviation followed the same half-Student-*t* distribution, that is,

$$\sigma \sim t_3^+(0, 2.5).$$

The correlation matrices of the random effects followed a Lewandowski–Kurowicka–Joe (LKJ) distribution with shape parameter 2,

$$\mathbf{R} \sim LKJ(2).$$

For generalized mixed effects models for cued recall and restudy decisions, priors were defined as follows:

Fixed effects for all non-intercept coefficients followed a Student-*t* distribution with 1 degree of freedom, mean 0, and scale 2.5, that is,

$$\beta_j \sim t_1(0, 2.5) \ .$$

The intercept followed a wider Student-t distribution with 1 degree of freedom, mean 0, and scale 10, that is,

$$\beta_0 \sim t_1(0, 10) \ .$$

Standard deviations of the random effects followed a half-Student-*t* distribution with 1 degree of freedom, mean 0, and scale 2.5, that is,

$$\sigma_k \sim t_1^+(0, 2.5),$$

and the correlation matrices of the random effects followed a Lewandowski–Kurowicka–Joe (LKJ) distribution with shape parameter 2,

$$\mathbf{R} \sim LKJ(2).$$

We relied on model comparisons to estimate Bayes Factors for non-intercept fixed effects. For main effects, we compared a model including main effects of all predictors with a model excluding the main effect of interest. For interaction effects, we compared the full model with a model including only main effects. We interpret Bayes factors based on the following conventions: < 1/10 = strong evidence for H_0; 1/10–1/3 = moderate evidence for H_0; 1/3–1 = anecdotal evidence for H_0; 1–3 = anecdotal evidence for H_1; 3–10 = moderate evidence for H_1; >10 = strong evidence for H_1 (van Doorn et al., 2021).

JOLs

Full results for the Bayesian linear mixed effects model for JOLs are presented in Table S2. Results converged with the frequentist linear mixed effects model in showing strong evidence for higher JOLs for related compared to unrelated pairs, $b = 40.24$, $BF_{10} = +\infty$.

Table S2

Bayesian Linear Mixed Model for JOLs Across Experiments 1a and 1b

Predictor	b	95% CI	BF_{10}
(Intercept)	47.33	[45.00, 49.58]	
Experiment	–0.44	[–3.93, 2.74]	0.79
Relatedness	**40.24**	**[37.08, 43.41]**	+∞

Note. Experiment (–0.5 = Experiment 1a, 0.5 = Experiment 1b) and Relatedness (–0.5 = unrelated pair, 0.5 = related pair) were entered as effect-coded predictors. *95% CI* refers to the 95% credibility interval for the effect estimate. *BF10* denotes the Bayes factor for the inclusion of each predictor based on model comparisons. We interpret Bayes factors based on the following conventions: < 1/10 = strong evidence for H_0; 1/10–1/3 = moderate evidence for H_0; 1/3–1 = anecdotal evidence for H_0; 1–3 = anecdotal evidence for H_1; 3–10 = moderate evidence for H_1; >10 = strong evidence for H_1 (van Doorn et al., 2021). Bold values indicate predictors whose 95% CI does not include 0.

Cued Recall

Full results for the Bayesian generalized mixed effects models for cued recall are presented in Table S3. Results converged with the frequentist linear mixed effects models in showing strong evidence for higher recall for related compared to unrelated pairs, $b = 2.38$, $BF_{10} = 1.21 \times 10^{63}$, and for an interaction effect between relatedness and judgment group, $b = 0.67$, $BF_{10} = 2{,}633.68$. Follow-up generalized mixed models indicated that this was due to no reactivity for related pairs, $b = 0.06$, $BF_{10} = 0.05$, but negative reactivity for unrelated pairs, $b = –0.63$, $BF_{10} = 107.94$, both supported by strong evidence.

Table S3

Bayesian Generalized Mixed Model for Cued Recall Across Experiments 1a and 1b

Predictor	(A) Related and unrelated pairs			(B) Related pairs			(C) Unrelated pairs		
	b	95% CI	BF_{10}	*b*	95% CI	BF_{10}	*b*	95% CI	BF_{10}
(Intercept)	0.40	[0.19, 0.61]		1.61	[1.37, 1.86]		–0.82	[–1.07, –0.57]	
Experiment	–0.09	[–0.40, 0.23]	8.77	–0.16	[–0.47, 0.16]	0.09	0.20	[–0.29, 0.69]	0.12
Judgment group	–0.28	[–0.57, 0.01]	2,383.97	0.06	[–0.25, 0.37]	0.05	**–0.63**	**[–0.96, –0.31]**	**107.94**
Relatedness	**2.38**	**[2.07, 2.68]**	$\mathbf{1.21 \times 10^{63}}$						
Judgment group × Relatedness	**0.67**	**[0.43, 0.92]**	**2,633.68**						

Note. Experiment (–0.5 = Experiment 1a, 0.5 = Experiment 1b), Judgment group (–0.5 = no JOL, 0.5 = JOL), and Relatedness (–0.5 = unrelated pair, 0.5 = related pair) were entered as effect-coded predictors. *95% CI* refers to the 95% credibility interval for the effect estimate. *BF10* denotes the Bayes factor for the inclusion of each predictor based on model comparisons. We interpret Bayes factors based on the following conventions: < 1/10 = strong evidence for H_0; 1/10–1/3 = moderate evidence for H_0; 1/3–1 = anecdotal evidence for H_0; 1–3 = anecdotal evidence for H_1; 3–10 = moderate evidence for H_1; >10 = strong evidence for H_1 (van Doorn et al., 2021). Bold values indicate predictors whose 95% CI does not include 0.

Restudy Decisions

Full results for the Bayesian generalized mixed effects models for restudy decisions are presented in Table S4. Results converged with the frequentist linear mixed effects models in showing strong evidence for less frequent decisions to restudy related compared to unrelated pairs, $b = -3.36$, $BF_{10} = +\infty$, and for an interaction effect between relatedness and judgment group, $b = 0.83$, $BF_{10} = 18.76$. Follow-up generalized mixed models, however, revealed no evidence for differences between groups in restudy decisions for related and unrelated pairs, as indicated by anecdotal evidence toward H0 for related pairs, $b = 0.54$, $BF_{10} = 0.46$, and moderate evidence toward H0 for unrelated pairs, $b = -0.35$, $BF_{10} = 0.22$.

Taken together, the Bayesian analyses of restudy decisions fully support conclusions derived from a frequentists approach by showing that restudy decisions for related and unrelated word pairs do not reliably differ by judgment group.

Table S4

Bayesian Generalized Mixed Model for Restudy Decisions Across Experiments 1a and 1b

Predictor	(A) Related and unrelated pairs			(B) Related pairs			(C) Unrelated pairs		
	b	95% CI	BF_{10}	*b*	95% CI	BF_{10}	*b*	95% CI	BF_{10}
(Intercept)	–1.73	[–2.02, –1.44]		–3.38	[–3.76, –3.01]		0.01	[–0.32, 0.33]	
Experiment	–0.30	[–0.83, 0.23]	0.20	–0.04	[–0.65, 0.58]	0.19	–0.62	[–1.28, 0.04]	0.40
Judgment group	0.06	[–0.46, 0.58]	0.07	0.54	[–0.06, 1.14]	0.46	–0.35	[–0.96, 0.27]	0.22
Relatedness	**–3.36**	**[–3.78, –2.95]**	∞						
Judgment group × Relatedness	**0.86**	**[0.17, 1.56]**	**18.76**						

Note. Experiment (–0.5 = Experiment 1a, 0.5 = Experiment 1b), Judgment group (–0.5 = no JOL, 0.5 = JOL), and Relatedness (–0.5 = unrelated pair, 0.5 = related pair) were entered as effect-coded predictors. *95% CI* refers to the 95% credibility interval for the effect estimate. *BF10* denotes the Bayes factor for the inclusion of each predictor based on model comparisons. We interpret Bayes factors based on the following conventions: < 1/10 = strong evidence for H_0; 1/10–1/3 = moderate evidence for H_0; 1/3–1 = anecdotal evidence for H_0; 1–3 = anecdotal evidence for H_1; 3–10 = moderate evidence for H_1; >10 = strong evidence for H_1 (van Doorn et al., 2021). Bold values indicate predictors whose 95% CI does not include 0.

References

Bürkner, P.-C. (2017). brms: An R package for bayesian multilevel models using stan. *Journal of Statistical Software*, *80*(1). https://doi.org/10.18637/jss.v080.i01

Gelman, A., Jakulin, A., Pittau, M. G., & Su, Y.-S. (2008). A weakly informative default prior distribution for logistic and other regression models. *The Annals of Applied Statistics*, *2*(4). https://doi.org/10.1214/08-AOAS191

van Doorn, J., van den Bergh, D., Böhm, U., Dablander, F., Derks, K., Draws, T., Etz, A., Evans, N. J., Gronau, Q. F., Haaf, J. M., Hinne, M., Kucharský, Š., Ly, A., Marsman, M., Matzke, D., Gupta, A. R. K. N., Sarafoglou, A., Stefan, A., Voelkel, J. G., & Wagenmakers, E.-J. (2021). The JASP guidelines for conducting and reporting a Bayesian analysis. *Psychonomic Bulletin & Review*, *28*(3), 813–826. https://doi.org/10.3758/s13423-020-01798-5
